# Comparing the vascular thromboembolic events following arteriovenous fistula in Chinese population with end-stage renal diseases receiving Clopidogrel versus Beraprost sodium therapy: a retrospective cohort study

**DOI:** 10.1186/s12882-018-1166-0

**Published:** 2018-12-27

**Authors:** Yu Zhou, Ling Du, Bo Tu, Qiquan Lai, Xiaonan Du, Bo Xu, Fan Zhang, Mingdong Zhao, Ziming Wan, Jiajie Lai

**Affiliations:** 1 Department of Nephrology, The First Affiliated Hospital of Chongqing Medical University, Youyi Road No.1, Yuzhong District, Chongqing, 400016 China; 2 Department of Anesthesiology, The Central Hospital of Wuhan, Tongji Medical College, Huazhong University of Science and Technology, Gusao Road No. 16, Jianghan District, Wuhan, 430014 Hubei China; 3 Department of Ultrasonography, The First Affiliated Hospital of Chongqing Medical University, Youyi Road No.1, Yuzhong District, Chongqing, 400016 China; 4 Department of Thoracic surgery, The First Affiliated Hospital of Sun Yat-sen University, Huangpu East Road No. 183, Huangpu District, Guangzhou, 510700 China; 5 Radiology Department, The First Affiliated Hospital of Sun Yat-sen University, Huangpu East Road No. 183, Huangpu District, Guangzhou, 510700 China; 6 Department of Orthopaedics, Jinshan Hospital, Fudan University, Longhang Road No. 1508, Jinshan District, Shanghai, 201508 China; 7 Department of Gynaecology and obstetrics, The First Affiliated Hospital of Sun Yat-sen University, Huangpu East Road No. 183, Huangpu District, Guangzhou, 510700 China

**Keywords:** Clopidogrel, Beraprost sodium, Vascular thromboembolic event, Arteriovenous fistula, Chinese population

## Abstract

**Background:**

To assess the time to first on-study vascular thromboembolic events (VTEs) of clopidogrel (CL) or beraprost sodium (BPS) in Chinese population with end-stage renal disease (ESRD) treated with arteriovenous fistula (AVF) surgery.

**Methods:**

From Jan 2009 to May 2015, 346 ESRD cases suffering an AVF surgery and undergoing oral administration of 75 mg CL (initial dose of 300 mg), 1 time/day, for 4 weeks or 40 μg BPS, 3 times/day, for 4 weeks were retrospectively assessed. The primary outcome was time to first on-study VTE.

**Results:**

In total, 222 ESRD cases (CL, *n* = 112; BPS, *n* = 110) were assessed, with a median follow-up time of 38.1 months (range, 37–40 months). The mean time to first on-study VTE was 1.2 weeks (0.5–2.3) and 1.8 weeks (1.2–3.8) for CL and BPS, respectively (HR 0.27, 95% CI 0.16–1.45; *P* = 0.00). An increased incidence of VTEs was found during the 1th-month follow-up, with rates of 14.2 and 5.5% for CL and BPS, respectively (*P* = 0.03). The difference persisted over time, with rates of 24.1 and 11.8% at final follow-up, respectively (*P* = 0.02).

**Conclusion:**

CL with an increased risk of VTEs tended to have a VTE within the 1st month after cessation compared with BPS.

## Background

Establishing and maintaining long-term vascular access is a prerequisite for hemodialysis in patients with end-stage renal disease (ESRD) [1–4]. An ideal vascular access should provide sufficient blood flow for hemodialysis to ensure the completion of hemodialysis procedures, as well as long enough use time and as few complications as possible, which is an important condition for sustaining long-term survival [5]. During the treatment of arteriovenous fistula (AVF), it is possible that anastomotic embolism can often lead to failure of fistula [6, 7]. Therefore, long-term dialysis patients have to face treatment trouble and mental pain [8, 9]. ESRD patients are considered at increased risk for vascular thromboembolic events (VTEs) after an AVF surgery [6]. The use of antiplatelet medications, specifically clopidogrel (CL) and beraprost sodium (BPS), is steadily increasing [10, 11]. Previous studies show that patients receiving either of these medications are at decreased risk for VTEs after an AVF surgery, but the risk in a large, generalizable Chinese population is unknown [12, 13]. To our knowledge, a direct comparison between CL and BPS has rarely been reported in the previous literature. Despite poor efficacy in ESRD patients, BPS and CL remain promising options to decrease AVF-related VTEs. Nevertheless, several unanswered questions remain.

The aim of this study was to assess the outcome of CL or BPS using time to first on-study VTEs as the primary endpoint in ESRD cases suffering an AVF surgery in Chinese population to further improve the design of the therapeutic regime.

## Methods

### Study population

All ESRD cases who were diagnosed according to standard criteria [14] and underwent an AVF surgery, following by peroral administration of 40 μg BPS(Beijing Ted Pharmaceutical Co., LTD., China, drug specifications: 20 μg), 3 times per day, for 1 month or 75 mg CL (initial dose of 300 mg, Hangzhou Sanofi Pharmaceutical Co., LTD., China, drug specifications: 75 mg), 1 time per day, for 1 month were identified from three medical centers (Jinshan Hospital, Fudan University; the First Affiliated Hospital, Sun Yat-sen University; the First Affiliated Hospital of Chongqing Medical University) between Jan 2009 and May 2015. Inclusion criteria: age ranging 20–50 years; within 4 weeks prior to surgery, no cardiovascular-related medications were applied in any case; dialysis was performed for 3 times/week, 3~4 h/time. Main exclusion criteria: chronic wasting disease; myelodysplastic syndrome; cardiovascular dynamic instability; coagulopathy; hemorrhagic endovasculitis; a long-term history of receiving the BPS or CL treatment; deterioration of renal function during follow-up; co-occurring tumour; Child-Turcotte-Pugh classification of C for liver function; clinical data which might lead to informative censoring for time to first on-study VTEs; loss of follow-up; interruption or modification of BPS or CL regimen; dropout owing to or severe infection; life expectancy less than 2 years; concomitant mental illness; an American Society of Anesthesiologists (ASA) score of IV or V. Patients would be censored at any time when they were loss to follow-up for the follow-up period, for whatever reason. Data collection for these cases was censored at completion of the study.

### Surgical technique

Preoperatively, the arteries with strong fluctuation and thick veins were marked. After local anesthesia, the longitudinal incision of the skin was performed in the view of the microscope for 1.5–2 cm, following by exposure, dissociation of cephalic vein and artery. The distal end of the cephalic vein was ligated. After the expansion of the proximal cephalic vein, the intravenous injection of heparin saline is used to test whether the cephalic vein is obstructed or not. The 45 ° Angle pruning of the anastomosis was performed. The peripheral membrane of the vascellum was clipped and the vascellum was repeatedly washed with heparin saline. The radial artery between the vascular clamps was distended by injection of heparin saline with a fine needle. The radial artery was opened longitudinally with a sharp knife, about 4–5 mm in length. End-to-side continuous anastomosis of the cephalic vein and radial artery was performed in line with relatively mature surgical techniques [15, 16]. Anastomoses were formed using 8.0 prolene. It should be noted that blood vessels should not be warped or subjected to pressure. During the suture process, the needle spacing should be as consistent as possible. After the completion of the anastomosis, the arterial clip was released and then clamped, followed by the intravenous clip. After 5 min, all the vascular clips were released. A small amount of blooding at the vascular anastomosis point can be gently pressed to stop the bleeding.

### Definitions of main descriptive variables

The primary endpoint was time to first on-study VTEs. Prior to the start of the study, an Endpoint Judgment Committee (EJC) that comprised of 3 independent ultrasound doctors who are not involved directly in the study was established in each institution. VTEs were defined as an interruption of blood flow owing to anastomosis-related stenosis or thrombus, which was determined by an EJC based on colour Doppler ultrasonography. Primary patency at the anastomoses was defined as the interval from the time of access to any intervention which involved in restoring or sustaining patency. Patency was censored by three independent ultrasound doctors at the date of on-study VTE survey. A venous diameter greater than 5 mm can be adapted to the need for dialysis and is not easily clogged. The success rate of internal fistula = patency number/total number *100%. The analysis of the rate for time to first and subsequent on-study VTEs is based on an Andersen-Gill model [17]. Major bleeding events were defined as prior statements [18].

### Statistical analysis

The data up to the primary analysis cut-off were valid data. Categorical variables were expressed as frequencies and percentages. Quantitative variables are presented as either the mean +/− standard deviation (SD) or median [interquartile range (IQR)] depending on the data distribution. Bivariate analyses involving ANOVA and Chi-square tests were applied as appropriate to assess between-group differences. The Kaplan-Meyer analysis were constructed to exhibit the incidence of the primary study endpoint. In computations of event rates, follow-up time was censored at lost to follow up or all-cause death. All- cause death was ascertained until the end date of the data cut-off. The Cox regression models was used to assess hazard ratio (HR). A *P* < 0.05 was considered significantly different. Statistical analysis is processed by IBM-SPSS (version 24.0, Inc., NY, USA).

## Results

### Baseline characteristics

**Fig. 1:**
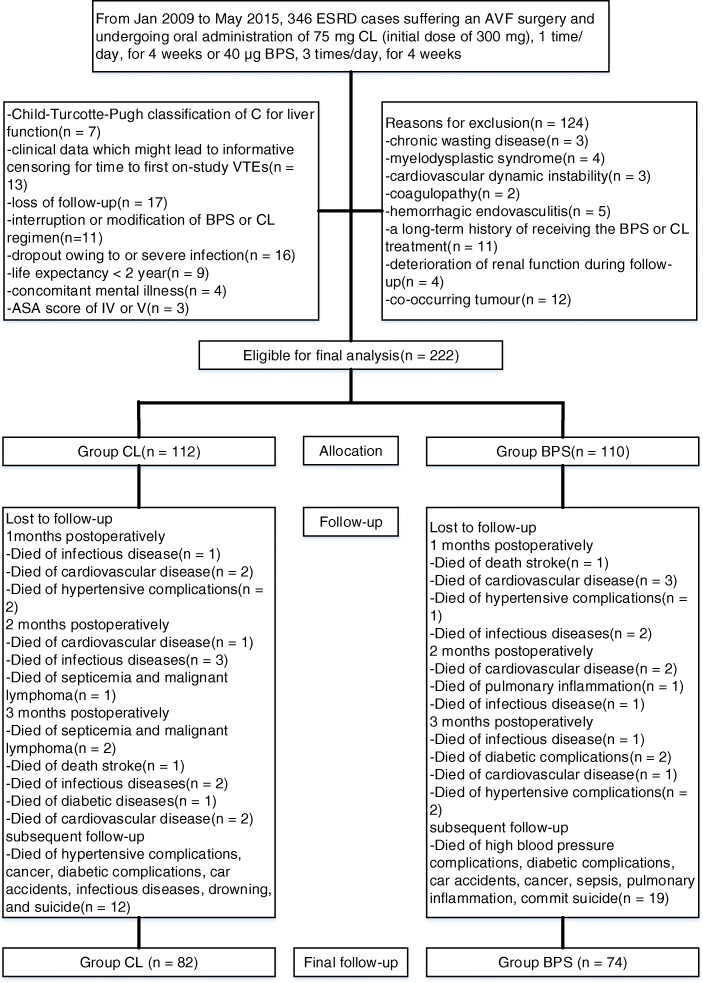
Flow diagram demonstrating methods for the population-based identification to assess the time to first on-study vascular thromboembolic events (VTEs) of clopidogrel (CL) or beraprost sodium (BPS) in Chinese population with end-stage renal disease (ESRD) treated with arteriovenous fistula (AVF)

During the study period, 346 consecutive ESRD cases who underwent an AVF surgery and received peroral administration of CL or BPS were identified. Of 346, 222 cases (CL, *n* = 112; BPS, *n* = 110) met criteria. Details are summarized in Fig. 1. Follow-up regarding the primary endpoint was completed in August 2016. Median follow-up time is 38.1 months (range, 37–40). Patient-related baseline data are showed in Table 1. No between-group significant differences were detected in medical related diseases following the initiation of drug intervention. Patient-related baseline characteristics were well balanced.

**Table 1:** Between-group comparison of baseline data

Variable	CL (*n* = 112)	BPS (*n* = 110)	*P* - value
Age (y)			0.73*^a^
20–39	35	33	
40–59	44	42	
60–79	33	35	
Sex, No. M/F	60/52	59/51	0.99*^b^
Systolic BP (mmHg)	167.13 ± 31.47	166.87 ± 34.79	0.15*^c^
Diastolic BP (mmHg)	102.52 ± 25.46	103.24 ± 23.47	0.22*^c^
Hypertension, No.	82	84	0.59*^b^
Hb (g/dL)	12.14 ± 1.35	12.36 ± 1.12	0.15^*c^
BUN (mg/dL)	43.37 ± 15.31	42.83 ± 17.68	0.16*^c^
eGFR (ml/min/1.73 m^2^)	13.15 ± 3.43	13.45 ± 3.62	0.48*^c^
Creatinine (mg/dL)	10.71 ± 1.48	10.42 ± 1.32	0.27*^c^
Dialysis duration with respective access (months)	14.22 ± 7.35	14.68 ± 7.22	0.18*^c^
Type dialysis, No.			0.37*^b^
Haemodialysis	107	102	
Peritoneal dialysis	5	8	
Ambulatory status			0.47*^a^
Normal walking	103	98	
Walking with assistive devices	9	12	
Completely restricted walking	0	0	
ASA level			0.94*^a^
1	31	34	
2	47	40	
3	34	36	
BMD	−2.57 ± 0.34	−2.49 ± 0.52	0.47*^c^
BMI (kg/m2)	24.71 ± 4.34	25.12 ± 4.61	0.36*^c^
Personal history of VTEs	16/112	18/110	0.67*^b^
Family history of VTEs	12/112	14/110	0.64*^b^
Diabetes mellitus			
Duration, year	19 (4–26)	18 (5–24)	0.14*^c^
Type 2, No.	27	29	0.70*^b^
Insulin use, No.	15	17	0.66*^b^

*CL* clopidogrel, *BPS* beraprost sodium, *BP* blood pressure, *Hb* haemoglobin, *BUN* blood urea nitrogen, *eGFR* estimated glomerular filtration rate, *ASA* American Society of Anesthesiologists, *BMD* bone mineral density, *BMI* body mass index, *VTEs* vascular thromboembolic events

*No statistically significant values

^a^Analysed using the Mann-Whitney test

^b^Analysed using the Chi-square test

^c^Analysed using an Independent-Samples t-test

### VTE incidence

The rates of the VTEs for the CL and BPS groups were 24.1% (27/112) and 11.8% (13/110), respectively (Table 2). The mean time to first on-study VTE was 1.2 weeks (0.5–2.3) and 1.8 weeks (1.2–3.8) for CL and BPS, respectively (HR 0.27, 95% CI 0.16–1.45; *P* = 0.00).

**Table 2:** Between-group comparison of VTE incidence

Variable	CL (*n* = 112)	BPS (*n* = 110)	*P* - value
Total incidence of VTEs	27/112	13/110	0.02*^a^
VTE incidence			
During the first Mos	16/112	6/110	0.03*^a^
During the second Mos	3/112	2/110	0.64^a^
from the third Mos to data cut-off	8/112	5/110	0.41^a^

*CL* clopidogrel, *BPS* beraprost sodium, *VTEs* vascular thromboembolic events, *Mos* month

*Statistically significant values

^a^Analysed using the Chi-square test

**Fig. 2:**
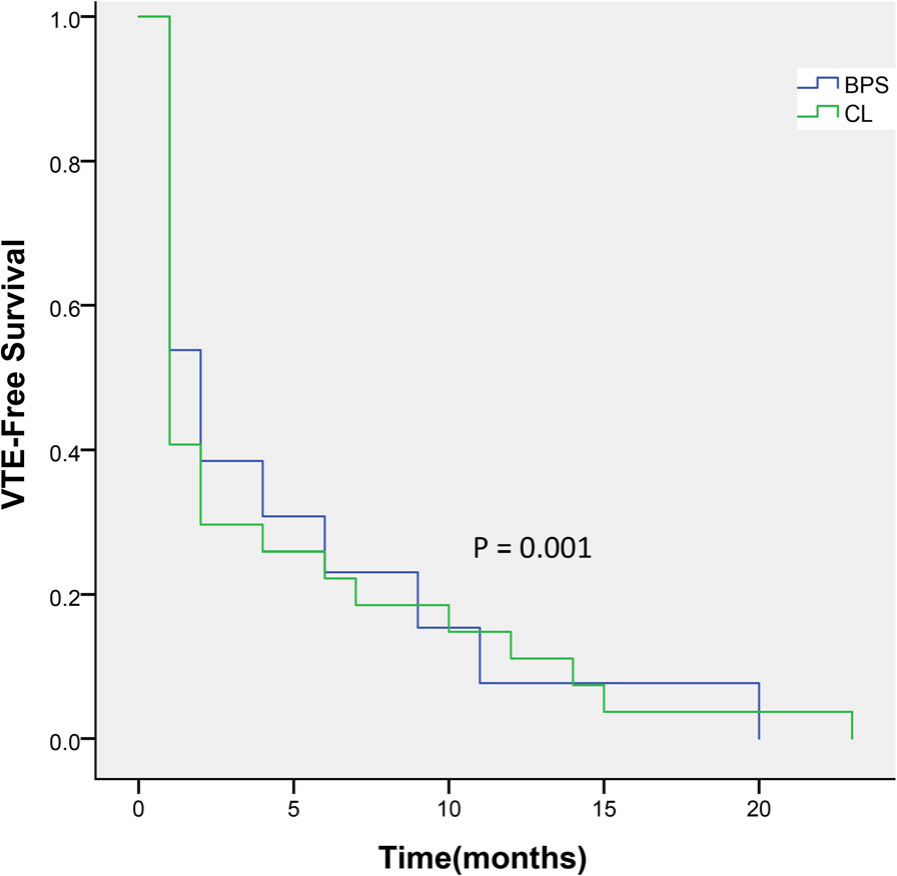
Kaplan–Meier estimates of vascular thromboembolic events (VTEs) of arteriovenous fistula (AVF) between groups. At the final follow-up, the incidence of VTEs was higher in the CL group than in the BPS group (*P* = 0.001 by Kaplan–Meier test). VTE-Free survival (time to first on-study VTE) comparing clopidogrel (CL) versus beraprost sodium (BPS)

CL tended to have a higher incidence of VTEs in comparison with BPS at final follow-up (27 vs. 13, respectively). During the 1st month after cessation, 16 (16/112) cases were diagnosed with a VTE; During the 2nd month, 3 underwent a VTE (3/112); Between 3rd month and data cut-off, 8 suffered a VTE (8/112). There is a distributed phenomenon of VTEs within the 1st month in the CL-treated cohort. However, it failed to be observed or was not apparent in the BPS-treated cohort. During the 1st month after cessation, 6 patients (3/110) had a VTE; during the 2nd month, 2 (2/110) underwent a VTE; from 3rd month to data cut-off, 5 (5/110) underwent a VTE. A decrease risk of VTEs by 47% was detected in BPS compared with CL (HR, 2.04; 95% CI 0.12–2.17; *P* = 0.001), as presented in Table 3 and Fig. 2.

**Table 3:** VTE risk ratio between groups

Variable	CL (*n* = 112)	BPS (*n* = 110)	*P* - value
Total VTE HR (95%CI)	6.98	3.42	0.001*
	(3.17-18.61)	(1.46–8.53)	
VTE HR (95%CI)			
during 1st Mos	7.15	3.29	0.135*
	(2.49-15.11)	(1.21–6.67)	
during 2nd Mos	26.58	24.37	0.181*
	(8.53-89.17)	(7.64–101.72)	
from 3rd Mos to data cut-off	19.36	14.15	0.214*
	(6.62-86.19)	(5.13–86.24)	

*CL* clopidogrel, *BPS* beraprost sodium, *VTE* vascular thromboembolic event, *HR* hazard ratio, *CI* confidence interval, *Mos* month

*Statistically significant values

### Adverse events

A major bleeding event happened among 5.4% of CL-treated cohort; and such event occurred in 4.5% of BPS-treated cohorts (*P* = 0.78). Compared with BPS, CL was not associated with an increased risk in major bleeding (HR1.06; 95%CI: 0.62–1.74). No significant differences were detected in other adverse events.

## Discussion

In the current study evaluating time to first on-study VTE among at-risk ESRD patients undergoing an AVF surgery, to our knowledge the largest AVF study to date, the major finding was that BPS demonstrated superiority to CL by decreasing the occurrence of VTEs (primary endpoint) in ESRD patients who underwent an AVF surgery. Notably, CL tended to result in a first on-study VTE within the 1st month after the cessation of drug administration compared with BPS.

At the study start, we hypothesized that excellent primary endpoint would be attributed to CL on account of several previous findings. Unfortunately, in our study, CL was inclined to lead to an increased risk of first on-study VTEs, which seemed to contradict its predictive efficacy of VTEs. The reasons for the finding remain indistinct and can be elaborated different versions in some recent studies in ESRD patients who underwent an AVF surgery [10, 11]. An explanation for the opposite finding is unhesitatingly unavailable, although CL which is commonly used to prevent ATEs might contribute to the difference [19]. The occurrence of VTEs remains a life-threatening clinical challenge, although considerable benefits of CL have been showed in previous clinical trials [20–22]. Thus far, several literatures have shown the negative impact of CL variability on VTEs in ESRD patients who underwent an AVF surgery, regardless of the prevention of heart attack and strokes in cardiovascular cases [23, 24]. Undeniably, our study underscored the potential CL-related risks that CL adopted in ESRD patients, which, compared with BPS, tended to be associated with a poor prognosis.

There is a body of evidence suggesting that poor vascular elasticity and endovascular stenosis underlie a significant proportion of patients of VTEs [25, 26]. Endometrial damage following an AVF surgery can contribute to the occurrence of VTEs [25, 27]. In terms of the success rate, there are many substantial studies indicated that BPS has contributed to the improvement of the success rate of AVFs in the ESRD patients compared with CL, although a finding at odds with trends was detected in several previous literatures [10, 28]. Nevertheless, the superiority of BPS has gained increasing recognition, and low VTEs in BPS is most likely explained by BPS itself [29]. Although the success rate of VTEs in our study was lower than anticipated, our finding was comparable to the outcome of a previous meta analysis [27]. Furthermore, consistent with previous studies [30, 31], high VTEs which were detected in CL-treated cohort were associated with an increased risk of thrombus complication.

Our finding is inconsistent with several reports on the rate of VTEs [4, 5, 32, 33]. A previous study by Yuo was a retrospective assessment only with a rather small number of cases, and the study failed to have a control group [30]. Furthermore, in their study the divergent results cannot be explained. A growing but still very limited body of literature has shown that BPS tended to result in VTEs compared with CL [19, 25]. This difference may be attributed to improved monitoring and management of VTEs. In addition, although VTEs were not detected in other cases with ESRD during the study, treatment in accordance with guidelines is advocated to manage VTE risk, and longer-term follow-up is necessary to ascertain any implications of the drug effect.

This study reflects the advantages of the relatively large sample size, which is inclined to provide statistical power to identify insignificant differences in the primary endpoint. The study excluded those cases with high risk for VTEs. Although such cases would be inclined to suffer a VTE, they also have a high rate of some other complications, which could result in high rates of withdrawal. Although our study has basically achieved the desired results, as a retrospective study that BPS is a more optimistic for ESRD patients in preventing VTEs compared with CL, there are inevitably some limitations. Firstly, between-study comparisons might tend to be confounded by differences in population included. Secondly, surgeon- and patient- related confounders may be inevitable. Nevertheless, improvement in the success rate of VTEs remained noteworthy, which had been proved by the final logistic regression assessment after adjusting some potentially and relatively imbalanced variates.

## Conclusions

The study demonstrates, as anticipated, the superiority of BPS over CL in ESRD patients using time to first on-study VTE as the primary study endpoint regardless of the follow-up period. Compared with CL, BPS shows a decreased risk for VTEs, the long-term significance of which remains to be clarified. However, the superior efficacy of BPS in the current study offers preliminary evidence of a benefit–risk that corroborates its application in ESRD cases receiving an AVF surgery.

## Abbreviations

ASA: American Society of Anesthesiologists
AVF: Arteriovenous fistula
BMD: Bone mineral density
BMI: Body mass index
BP: Blood pressure
BPS: Beraprost sodium
BUN: Blood urea nitrogen
CI: Confidence interval
CL: Clopidogrel
eGFR: Estimated glomerular filtration rate
EJC: Endpoint Judgment Committee
ESRD: End-stage renal disease
Hb: Haemoglobin
HR: Hazard ratio
IQR: Interquartile range
Mos: Month
SD: Standard deviation
VTEs: Vascular thromboembolic events
